# Trophic Transfer of Arsenic from an Aquatic Insect to Terrestrial Insect Predators

**DOI:** 10.1371/journal.pone.0067817

**Published:** 2013-06-27

**Authors:** Christina L. Mogren, William E. Walton, David R. Parker, John T. Trumble

**Affiliations:** 1 Department of Entomology, University of California Riverside, Riverside, California, United States of America; 2 Department of Environmental Science, University of California Riverside, Riverside, California, United States of America; Dowling College, United States of America

## Abstract

The movement of energy and nutrients from aquatic to terrestrial ecosystems can be substantial, and emergent aquatic insects can serve as biovectors not only for nutrients, but also for contaminants present in the aquatic environment. The terrestrial predators *Tenodera aridifolia sinensis* (Mantodea: Mantidae) and *Tidarren haemorrhoidale* (Araneae: Theridiidae) and the aquatic predator *Buenoa scimitra* (Hemiptera: Notonectidae) were chosen to evaluate the efficacy of arsenic transfer between aquatic and terrestrial environments. *Culex tarsalis* larvae were reared in either control water or water containing 1000 µg l^−1^ arsenic. Adults that emerged from the control and arsenic treatments were fed to the terrestrial predators, and fourth instar larvae were fed to the aquatic predator reared in control or arsenic contaminated water. *Tenodera a. sinensis* fed arsenic-treated *Cx. tarsalis* accumulated 658±130 ng g^−1^ of arsenic. There was no significant difference between control and arsenic-fed *T. haemorrhoidale* (range 142–290 ng g^−1^). *Buenoa scimitra* accumulated 5120±406 ng g^−1^ of arsenic when exposed to arsenic-fed *Cx. tarsalis* and reared in water containing 1000 µg l^−1^ arsenic. There was no significant difference between controls or arsenic-fed *B. scimitra* that were not exposed to water-borne arsenic, indicating that for this species environmental exposure was more important in accumulation than strictly dietary arsenic. These results indicate that transfer to terrestrial predators may play an important role in arsenic cycling, which would be particularly true during periods of mass emergence of potential insect biovectors. Trophic transfer within the aquatic environment may still occur with secondary predation, or in predators with different feeding strategies.

## Introduction

Numerous studies have evaluated the effects of runoff on nontarget aquatic life [1]. Urban runoff [2] and erosion [3], [4] have been considered important routes for heavy metal and metalloid contaminants, such as arsenic, to reach aquatic systems. However, more recently, it has become apparent that the flow of nutrients and contaminants is not unidirectional, and that energetic pathways also link aquatic to terrestrial systems [5]. In aquatic-to-terrestrial transport of contaminants, the movements of animals that have accumulated contaminants, either through space or through trophic transfer, allows for the transfer of potentially harmful substances to locations away from the contaminant origin [6]. Aquatic insects, whose biomass can account for up to 190 kg ha^−1^ d^−1^ in very productive lake systems [7], have been shown to effectively export contaminants such as anthropogenic nitrogen and polychlorinated biphenyls from aquatic systems to terrestrial predators [8], [9], [10].

Arsenic is a widespread surface and ground water contaminant worldwide [11]. The terrestrial bogong moth, *Agrotis infusa* (Lepidoptera: Noctuidae), has been shown to act as a biovector of arsenic when migrating individuals estivate gregariously [12]. In the environment, arsenic may result from both natural and anthropogenic sources. Natural sources of arsenic in soils are mainly the parent materials from which arsenic is derived and volcanic activity, while anthropogenic sources include lead-arsenate insecticides, irrigation, and atmospheric deposition resulting from the burning of fossil fuels and copper smelting [3]. Weather events and runoff then transfer arsenic into aquatic systems.

In the United States, the Environmental Protection Agency (EPA) regulates safe concentrations of arsenic in surface waters for aquatic life, given its designation as a priority toxic pollutant. The maximum safe concentration for chronic exposure is 150 µg l^−1^ [13], although concentrations can exceed 1000 µg l^−1^ [11]. Concentrations in soils typically average 1–40 mg kg^−1^ and can exceed 600 mg kg^−1^ in contaminated locations ([14] and references therein). The forms most often encountered in the environment are arsenate, which substitutes for phosphate in ATP synthesis, and arsenite, which has a high affinity for sulfhydryl bonds and disrupts protein folding [15]. While the abiotic processes linking terrestrial and aquatic arsenic cycles have been extensively studied [3], [4], the ecological interactions of biovectors and arsenic cycling pertaining to insects is not well understood. The trophic transfer of arsenic between an invertebrate prey and insect [16]–[19] or vertebrate [20], [21] predators has been documented for aquatic systems. However, in terrestrial systems, trophic transfer of arsenic has only been evaluated for movement to vertebrate predators [22]–[26].

The goal of this research is to evaluate the potential for trophic transfer of arsenic from the aquatic to terrestrial environment via insects, and to evaluate efficiency of transfer for three aquatic or terrestrial predatory species with different feeding strategies that prey on mosquitoes. For this purpose we chose the Chinese praying mantis, *Tenodera aridifolia sinensis* (Mantodea: Mantidae), which is a widespread mantid native to east Asia that is often used in behavior and physiology studies [27]; the orb-weaving spider, *Tidarren haemorrhoidale* (Araneae: Theridiidae), a native to southern California and known to feed on small flying insects [28], and; the backswimmer, *Buenoa scimitra* (Hemiptera: Notonectidae), which flies nocturnally (thus occasionally leaving the aquatic system [29]), and is an effective predator of mosquito larvae being evaluated for release as a biological control agent against mosquitoes [30]. The ease in rearing these predators combined with their accessibility makes them optimal laboratory assay organisms.

## Methods

### Mosquito Rearing

Mosquito egg rafts of *Cx. tarsalis* were obtained from colonies maintained at the University of California, Riverside. Eggs were hatched in shallow white enamel pans (39×23×10 cm or 39×23×6 cm) containing 3 liters of tap water. Arsenic-treated water contained 1000 µg l^−1^ of sodium hydrogen arsenate heptahydrate, 99.998% (Sigma-Aldrich, St. Louis, MO, USA). This concentration was chosen because it represents a high yet still ecologically relevant concentration of arsenic [11], [31]. Pans were kept in an environmental rearing chamber under a 16∶8 L:D cycle at 28.0±0.25°C, and larvae were fed a mixture of a 3∶1 (wt:wt) ground mouse chow (mouse/rat diet, Harlan/Teklad, Madison, WI, USA) and brewer’s yeast (MP Biochemicals, Aurora, OH, USA) as a 10% suspension in deionized water.

At approximately one week after hatching, pupae were removed using a mesh net and transferred to a bowl of deionized water. The bowl was placed inside a 30 cm×30 cm×30 cm plastic insect cage at ambient light and temperature for adult emergence. Adults were provided with a 20% sucrose solution for feeding [32]. Larvae of *Cx. tarsalis* accumulated 6200±397 ng g^−1^ of arsenic and adults retained 2450±242 ng g^−1^.

### Arsenic Analysis

All of the arsenic analyses were conducted on entire individual insects using a previously described method [31], [33]. Briefly, samples underwent a two step microwave digestion process with sodium persulfate, sodium fluoride (Sigma-Aldrich, St. Louis, MO, USA), and nitric acid in HP-500 Teflon PFA digestion vessels (CEM Corporation, Matthews, NC, USA). Once cooled, the digestate was diluted and an aliquot pre-reduced using concentrated HCl and a 5%/5% w/w KI (potassium iodide, Sigma-Aldrich, St. Louis, MO, USA)/L-ascorbic acid (Fisher Scientific, Pittsburgh, PA, USA) solution. Arsenic concentrations were detected using a Perkin-Elmer (Waltham, MA, USA) Analyst 800 Atomic Absorption Spectrophotometer. The minimum detection limit of the HGAAS was previously determined to be 0.050 µg l^−1^ for arsenic [31]. Simultaneous digestions of oyster tissue standard reference material (NIST 1566b, Gaithersberg, MD, USA) and reagent blanks were conducted to validate the arsenic concentrations recovered in the unknown insect tissues.

### Tenodera Aridifolia Sinensis

Egg masses of *Tenodera aridifolia sinensis* were purchased from Rincon-Vitova Insectaries (Ventura, CA) and hatched in 5-liter aquaria covered with cheesecloth in an environmental rearing chamber at 28°C and 16L:8D light cycle. A beaker containing water was placed inside the aquaria to increase humidity and induce hatching. A branch of *Photinia* sp. was also added to reduce cannibalism of newly hatched first instars. The eggs hatched two weeks after arriving. Once hatched, each of 40 individuals were transferred to a 600 ml beaker containing a moist cotton ball and covered with cheesecloth, similar to Kaltenpoth [34]. Twenty nymphs were randomly assigned to the control treatment (fed adult control mosquitoes) and the other 20 were assigned to the arsenic treatment (fed adult mosquitoes exposed to 1000 µg l^−1^ arsenate as larvae).

Mantids were provided with one mosquito from their respective treatment group after hatching, and within 2 hours, most of the first instars had caught and eaten the mosquitoes. While first instars, each individual *T. a. sinensis* was fed one mosquito daily. Second instars were fed two mosquitoes daily and third instars were fed three mosquitoes daily. If a mosquito died before being consumed, then it was replaced. Once individuals reached the fourth instar (approximately 31 days), they were fed three more mosquitoes and then allowed to depurate for 48 hours before being frozen and analyzed for arsenic. Instar, the number of mosquitoes fed, the number of mosquitoes consumed, and survival were monitored daily. Morphometric parameters were measured (head width, hind tibia length, body length, dry mass, longevity). Individuals were then oven dried, a final dried mass recorded, and digested and analyzed for arsenic accumulation.

Multiple regression analysis using backwards model selection (SAS v.9.2) was used to determine if any of the morphometric parameters, in addition to treatment and total number of mosquitoes consumed, were significant predictors of the final arsenic concentration. Although arsenic data were normally distributed, they did not uphold the assumption of homogeneous variances, and thus data were square root transformed. Beakers were blocked by location and a Relative Growth Index (RGI) calculated ([35], after [36]). Abbott’s formula [37] was applied to correct for control mortality. Significant differences between treatments were determined using repeated measures ANOVA in R Statistical Software (v.2.15.0) with the lme4 package.

### Tidarren Haemorrhoidale

Forty individuals of *T. haemorrhoidale* were field collected from the University of California Riverside Botanic Garden in Riverside, California from a stand of *Opuntia littoralis* var. *vaseyi*, a prickly pear cactus native to southern California. Because *T. haemorrhoidale* were field-collected, a subset of individuals were sacrificed prior to the start of the experiment and analyzed for arsenic that may have been accumulated from the environment. Arsenic was not detected in *T. haemorrhoidale* using HGAAS.

A preliminary mass was taken before individuals were randomly assigned to a treatment group, 20 controls and 20 for the arsenic treatment. Individuals were placed in a 600 ml beaker and provided with a twisted piece of wire covered with masking tape as a web-building substrate. Beakers were covered with cheese cloth. Spiders were allowed to depurate for one week before being fed their respective treatment mosquitoes at a rate of one every three days. This interval was deemed appropriate because in preliminary tests, individuals would not feed daily and mosquitoes would die before being consumed. The depuration period gave the spiders time to construct a web and once mosquitoes were caught in the web, the spiders were observed wrapping them in silk and feeding upon them.

The experiment was terminated after 30 days and the spiders preserved in ethanol. The species was verified using Levi [38] and all specimens were determined to be adult females. A final mass was taken and the total number of consumed mosquitoes tallied (carcasses wrapped in silk were cast from the web after feeding). The total number of fecal spots, which measured 1–4 mm in diameter, was also recorded. Individuals were then oven dried, a final dried mass recorded, and then digested and analyzed for arsenic accumulation.

An analysis of covariance (ANCOVA) (SAS v.9.2) was used to determine if population means of the final mass (dependent variable) were the same across treatments (independent variable), while controlling for initial mass. Multiple regression analysis using backwards model selection (SAS v.9.2) was used to determine if any of the measured variables affected arsenic accumulation in *T. haemorrhoidale* adult females. A square root transformation was applied to achieve normality and homogeneous variances. Abbott’s formula was applied to correct for control mortality [37]. Morphometric measurements were not possible for *T. haemorrhoidale* because all of the individuals used were adults, and they could not be reared from a standardized age.

### Buenoa scimitra

Adults of *Buenoa scimitra* were collected from the Valley Sanitary District Treatment Wetland C in Indio, CA (access was authorized by the VSD for insect collection as part of an ongoing mosquito monitoring partnership with W.E.W.). The temperature in the wetland averages 21.8±0.7°C, pH averages 7.8±0.1, and dissolved oxygen averages 5.4±1.6 mg l^−1^. The average concentrations of ammonium-nitrogen and nitrate-nitrogen are 41.5±4.30 and 5.1±1.3 mg l^−1^, respectively (Walton, unpublished data). Inductively Coupled Plasma Optical Emission Spectrometry (ICP-OES) (Perkin-Elmer Optima 7300 DV, Waltham, MA, USA) analysis of the wetland water revealed that arsenic levels were not detectable. The notonectid species was verified and voucher specimens deposited in the UCR Entomology Research Museum collection (UCRC ENT 145417 and 145418). Because *B. scimitra* adults were field-collected, a subset of individuals were digested and analyzed using HGASS to determine if arsenic was present prior to the start of the experiment. While low levels of arsenic were detected in individuals from Wetland C (102±69.9 ng g^−1^), they were significantly lower than those in the control treatment, and thus did not affect experimental outcomes.

Individuals were transported back to the lab and transferred to a 37.9-liter aquarium containing 50% tap and 50% deionized water and reared following the procedures of Hazelrigg [39]. However, because *B. scimitra* is a free swimming notonectid, no substrate was provided. Eight individuals were transferred to each of fifteen 9.5-liter aquaria filled with 6 liters of tap water and pre-rinsed quartz sand (Repti Sand, Zoo Med Laboratories, Inc., San Luis Obispo, CA, USA). The aquaria were randomly assigned to one of three treatments: control water with control mosquitoes (0/0), control water with arsenic reared mosquitoes (0/As), or arsenic water and arsenic reared mosquitoes (As/As), with five replicate aquaria per treatment. These aquaria were covered and aerated in a rearing room. Water temperature was 23.3°C through the duration of the experiment.

The notonectids were allowed to depurate for 48 hours before arsenic was added to the VV treatment (as sodium hydrogen arsenate heptahydrate, 99.998%, Sigma-Aldrich, St. Louis, MO, USA). After this point, each treatment received an input of approximately 40 mosquitoes daily for a month. This feeding rate was deemed appropriate because the following day, there would only be a couple mosquito larvae left. The notonectids are voracious mosquito predators [30], and as soon as the mosquitoes were introduced, they were observed to strike at and feed on the larvae. Survival of notonectids was monitored daily. After 30 days, the notonectids from each treatment replicate were frozen and then oven dried. A dry mass was taken prior to digestion and arsenic analysis.

Multiple regression analysis using backwards model selection (SAS v.9.2) was used to determine if treatment, treatment replicate, or mass were good predictors of the total arsenic concentration in individuals. Treatment replicate was included as a variable to ensure there were no significant differences between replicates. A double square root transformation was applied to achieve normality and homogeneous variances. Abbott’s formula was applied to correct for control mortality [37]. Morphometric measurements were not possible for *B. scimitra* because all of the individuals used were adults, and they could not be reared from a standardized age.

## Results

### Arsenic Analysis

Digestion and prereduction blanks (containing only prereduction solutions and 6 ml Milli-Q HPLC-grade water) revealed no major sources of arsenic contamination from the reagents used or from analyst error, and any arsenic present was below detection limits (0.050 µg l^−1^). Digestion and analysis of the NIST oyster tissue validated the protocol used to recover and analyze arsenic from the insect tissues with recoveries from the *T. a. sinensis*, *T. haemorrhoidale*, and *B. scimitra* digestions of 102±3.67%, 105±3.07%, and 110±1.92%, respectively. These values are all in agreement with previously published recovery values [31]. ICP-OES analysis of the mosquito rearing water and the water *B. scimitra* were reared in revealed that actual arsenic concentrations were within 7% agreement of the target concentration of 1000 µg l^−1^ in the arsenic treatments. 11±0.00 µg l^−1^ of arsenic was detected in control water, but this is attributed to arsenic being present at safe drinking levels in the tap water in which insects were reared.

### Tenodera Aridifolia Sinensis

Results from multiple regression analysis indicated that of the seven independent variables considered (treatment, instar, lifespan, headwidth, tibia length, body length, and number of mosquitoes consumed), treatment, instar, and lifespan were good predictors of final arsenic concentration in *T. a. sinensis* (AIC = 409), although the variable instar was removed because of multicollinearity (VIF = 4.23), and thus the analysis was conducted using only treatment and lifespan. There was no significant interaction between these two variables (df = 1,33, F = 0.37, p = 0.547). However, there was a significant effect of treatment on arsenic accumulation (Table 1). The amount of arsenic accumulated by individuals did not, however, significantly affect RGI (df = 1, χ^2^ = 0.064, p = 0.801). There was also no significant treatment effect on lifespan or morphometric characters, and there was no significant difference between treatments for the number of mosquitoes consumed (Table 2).

**Table 1:** Arsenic accumulation in the terrestrial predators.

	Control			As(V)			t	P
	N	Mean	95% CI	N	Mean	95% CI		
*Tenodera aridifolia sinensis*	15	145	63.4 – 227	19	658	384–931	4.54	<0.001
*Tidarren haemorrhoidale*	13	142	20.7–264	17	291	104–478	1.46	0.158

Units are ng g^−1^ dry weight. The arsenic treatments were fed *Cx. tarsalis* adults that retained 2450±242 ng g^−1^.

**Table 2:** Lifespan, morphometric characteristics, and number of mosquitoes consumed for *Tenodera aridifolia sinensis*.

	Control			As(V)			F	P
	N	Mean	SE	N	Mean	SE		
Lifespan (d)	17	35.5	1.02	20	35.3	1.33	0.02	0.899
Tibia length(mm)	17	7.76	0.16	20	7.55	0.22	0.59	0.447
Headwidth(mm)	17	3.44	0.09	20	3.43	0.08	0.00	0.966
Body length(cm)	17	2.50	0.07	20	2.40	0.08	0.78	0.382
Mosquitoes consumed	17	45.1	2.21	20	46.4	2.78	0.12	0.727

There were no significant differences between treatments.

Samples of mantid frass, liquid excrement, and exoskeletons were analyzed at the Stanford Synchrotron Radiation Lightsource (Menlo Park, CA) using x-ray absorption spectroscopy (XAS) (Mogren, unpublished data). Any arsenic that was being excreted through these mechanisms by *T. a. sinsensis* was below detection limits, indicating that these insects may not efficiently excrete arsenic and will therefore accumulate dietary arsenic in their bodies.

### Tidarren haemorrhoidale

ANCOVA revealed no significant covariate effect of the initial mass of the spiders with the final mass of the spiders between treatments, and no effect of treatment on the final mass of *T. haemorrhoidale* (control: 12.0±0.59 mg; As: 11.5±0.58 mg, F = 2.10, df = 2,30, p = 0.140). Results from multiple regression analysis indicated that of the five independent variables (treatment, initial mass, final mass, cast prey, and number of fecal spots) examined, treatment, final mass, and number of fecal spots were good predictors of the final arsenic concentration (AIC = 198). There were no significant interactions between these variables (p>0.05 for all 2-way combinations), and no multicollinearity (average VIF = 1.05). There was no significant difference in As accumulation between control-fed and arsenic-fed spiders (Table 1). Similarly, there was no significant effect of treatment on the number of fecal spots (a measure of egestion and potential detoxification) produced by individuals during the course of the experiment (control: 14.4±1.46 fecal spots; As: 13.9±1.02 fecal spots; df = 1,33, F = 2.50, p = 0.126). There was no significant treatment effect on the number of cast prey (control: 13.4±1.82 cast prey; As: 13.0±2.18; df = 1,33, F = 1.10, p = 0.302).

### Buenoa scimitra

**Figure 1:**
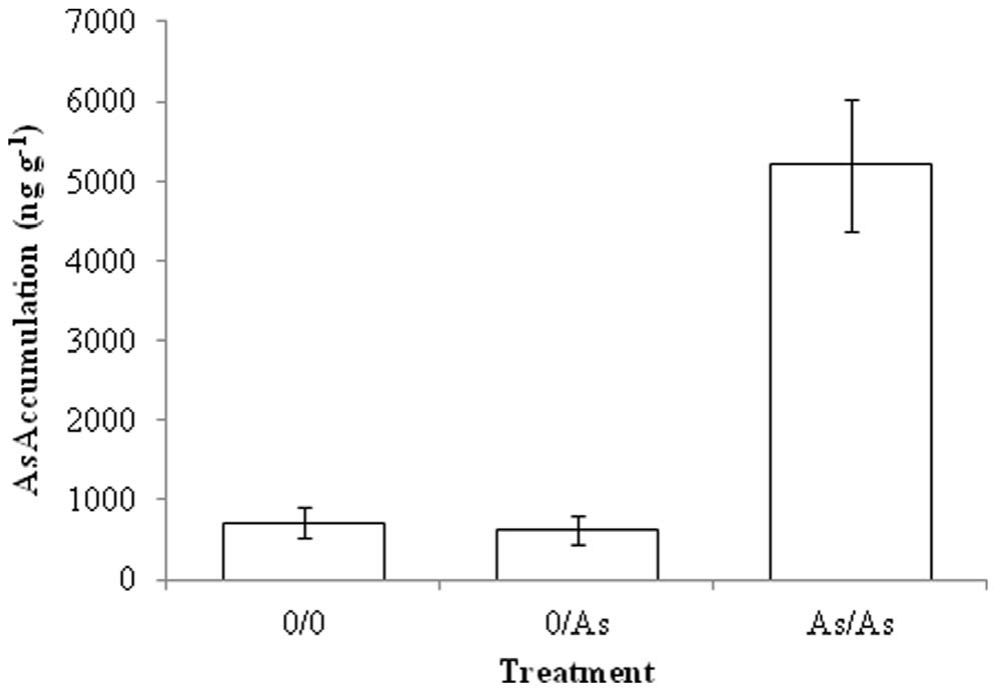
Arsenic accumulation in the aquatic predator, *Buenoa scimitra*. Mean ±95% CI. N = 35 for the 0/0 treatment (control water and control mosquitoes), N = 28 for the 0/As treatment (control water and arsenic-fed mosquitoes), and N = 34 for the As/As treatment (arsenic water and arsenic-fed mosquitoes). The arsenic treatments were fed fourth instar *Cx. tarsalis* prey that accumulated 6200±397 ng g^−1^.

Results from multiple regression analysis indicated that only the variable treatment was a good predictor of the final arsenic concentration, thus differences between treatments were analyzed using a one-way ANOVA. There was a significant difference between the three treatments (0/0, 0/As, As/As) (df = 2,94, F = 164, p<0.001; Figure 1) and Tukey’s post hoc comparisons showed the As/As treatment having significantly greater amounts of arsenic than both the 0/0 and 0/As treatments (p<0.001), which were not significantly different from each other (p = 0.644). This indicates that water contamination may play a more important role than prey ingestion in arsenic accumulation for these predators. There was a high degree of variability in the As/As treatment, and outliers as high as 40,000 ng g^−1^ were excluded from analysis (two outliers were removed based on studentized residuals greater than 2 [40]).

## Discussion

The terrestrial predators evaluated for trophic transfer in this experiment have different feeding strategies: spiders feed by injecting digestive fluids into their prey and ingesting the partially digested remains, while mantids consume prey whole. Therefore, any indigestible parts of the prey that may contain arsenic, such as the prey’s exoskeleton, would not be biologically available to the spider. This may explain the significant difference observed in arsenic accumulation for *T. a. sinensis* and *T. haemorrhoidale*, where *T. a. sinensis* accumulated twice as much arsenic as *T. haemorrhoidale* when fed adults of *Cx. tarsalis*. Previous work using X-ray Absorption Spectroscopy (XAS) has shown that in adults of *Cx. tarsalis*, arsenic is accumulated mostly in the exoskeleton of the thorax [41], which would be largely unavailable to a piercing-sucking predator. However, *B. scimitra*, which also has a piercing-sucking feeding strategy, accumulated eight times more arsenic in the As/As treatment than *T. a. sinensis* and 18 times more arsenic than *T. haemorrhoidale*. Increased accumulation in this species may be due to the larvae of *Cx. tarsalis* having more than twice as much accumulated arsenic as adults, or because *B. scimitra* was also exposed to arsenic through the water. At least in this case, direct environmental exposures play a much more significant role in accumulation than dietary exposure.

Other aquatic predators have also been shown to accumulate arsenic through trophic exposure. In a field study, *Sialis velata* (Megaloptera: Sialidae) only accumulated arsenic from contaminated chironomid prey, and the presence of arsenic in water did not affect overall accumulation in *S. velata* [16], in contrast to our findings with *B. scimitra*. Burghelea et al. [17] reported three species of predaceous dystiscids living in water containing 0.32 µg l^−1^ of arsenic accumulating 320±40 ng g^−1^ (*Hydroglyphus pusillus*), 410±100 ng g^−1^ (*Laccophilus minutus*), and 320±50 ng g^−1^ (*Rhantus suturalis*). However, Lavilla et al. [18] found that 98% of total arsenic in three species of anisopterans was bound to the exoskeleton as opposed to being ingested and accumulated (see also [42]). The differences observed in accumulation and trophic transfer of arsenic from prey to predators in these field experiments and the results from *B. scimitra* may be due to an interplay of biotic and abiotic factors (water chemistry, fluctuations in flow) in the field that cannot be replicated in the lab [19], and which may affect transfer through the food web. The ability of a specific aquatic predator to accumulate arsenic will likely be affected by the physiology of that predator and the accumulation and biotransformation abilities of the prey, in addition to abiotic conditions [42].

The trophic transfer literature with regards to terrestrial systems focuses mainly on the effects of arsenic on vertebrates. Banded water snakes fed fish from a contaminated location did not exhibit signs of physiological stress, though they did accumulate arsenic [22]. However, potential effects of arsenic were confounded by the presence of many other elements, and how arsenic moved through the food chain can only be inferred because invertebrates were not sampled from the location. Arsenic accumulation was similarly negligible in mice living in a contaminated seasonal wetland, whose diet consisted of seeds, plants, and arthropods that accumulated significant concentrations of arsenic (up to 0.419 mg kg^−1^ in arthropod prey) [23]. In contrast, studies addressing the affects of organoarsenical pesticides on woodpeckers and their prey, the mountain pine beetle, found that the birds accumulated potentially toxic levels of arsenic from the beetles [24], and that these levels caused both lethal [25] and sublethal [26] effects. The much more toxic effects of arsenic moving up the food chain in these studies may be the result of the organic form of arsenic in which beetles and birds were exposed versus inorganic forms in the Hopkins et al. [22] and Torres and Johnson [23] studies, as well as the concentrations accumulated in the mountain pine beetles. Differences between these vertebrate predators and the invertebrate predators *T. a. sinensis* and *T. haemorrhoidale* may be attributed to exposure levels and the form of arsenic in which the predators are ultimately exposed. The degree of arsenic accumulation may be further influenced by the detoxification and excretion pathways within the insect predators, which are thought to be mediated by glutathione [43], [44], [45].

Examples of arsenic trophic transfer with a terrestrial insect as the biovector have been shown for the bogong moth, *Agrotis infusa*. The gregarious estivations of this moth, which reach densities in the millions, result in the concentrating of arsenic to phytotoxic levels [12]. Although a direct correlation of trophic transfer to vertebrate predators has yet to be documented in this system, there is evidence for accumulation of arsenic in birds and small mammals in this system, with small predators accumulating more arsenic than herbivores [46].

Within an aquatic environment, arsenic is not likely to biomagnify [20], [21], [47], though it is bioavailable and does accumulate in invertebrates, as seen in *B. scimitra*. With regards to the linkages between aquatic and terrestrial arsenic cycles, there are no published field studies investigating the movement of this contaminant via emergent insects, or the effects they have on terrestrial predators, although there is theoretical evidence that inputs could be substantial [5], [9]. However, both mantids [48] and spiders [49] are shown to ingest insects with aquatic life stages in the wild. Recent work suggests that energy flows from aquatic to terrestrial environments may positively affect productivity in adjacent habitats through the rapid incorporation of insect detritus [50], [51]. However, there could also be negative consequences to these inputs when insects are exposed to persistent environmental contaminants, such as organic polychlorinated biphenyls (PCBs) [10] and heavy metals such as mercury [52]. Even if individuals emerging from the aquatic environment do not contain heavy loads of arsenic, the potential for the biomagnification of arsenic from mass emergences to adjacent terrestrial habitats is high [6]. Some aquatic insects, such as notonectids, are known to disperse en masse to different aquatic habitats, and could therefore vector contaminants from aquatic to aquatic systems as well [18], [53].

Further research is needed to evaluate the transfer potential of arsenic to terrestrial systems by emergent aquatic invertebrate biovectors, particularly in the context of climate change, as warmer temperatures may allow for more generations of consumers to be produced, increasing flow to terrestrial systems. Similarly, studies evaluating the movement of arsenic within aquatic food webs would provide valuable links in the biotic transfer potential of arsenic. Based on the results from this study, the generalist predator *T. a. sinensis* has the potential to be a good organism for tracking the movement of arsenic from aquatic to terrestrial predators, and other generalist terrestrial predators, such as ground beetles, should be evaluated as well.
